# User groups of inpatient multidisciplinary therapies for Parkinson’s disease in Germany: a bicenter prospective observational study

**DOI:** 10.1186/s42466-025-00409-9

**Published:** 2025-07-28

**Authors:** Vera Tschentscher, Judith Oppermann, Julius Welzel, Johanna Geritz, Ralf Gold, Siegfried Muhlack, Clint Hansen, Walter Maetzler, Lars Tönges, Raphael Scherbaum

**Affiliations:** 1 Department of Neurology, St. Josef-Hospital, Ruhr University Bochum, 44791 Bochum, Germany; 2 Department of Neurology, Kiel University, 24105 Kiel, Germany

**Keywords:** Parkinson's disease, Multidisciplinary, Inpatient, Gait, Wearable devices, Health services

## Abstract

**Background:**

In Germany, multidisciplinary care for people with Parkinson's disease (PwP, PD) is mainly provided in the inpatient setting. Differences in user groups between established and effective interventions like PD Multimodal Complex Therapy (PD-MCT) and Geriatric Complex Therapy (GCT) have not been investigated.

**Methods:**

This real-world bicenter prospective observational study involved PwP undergoing 14-day inpatient multidisciplinary therapies at two German university hospitals providing either PD-MCT or GCT. Demographic and clinical variables were recorded before and device-based gait variables before and after therapy. Non-parametric and parametric tests including ANCOVA with age as covariate were conducted to compare groups at baseline, and an exploratory binomial logistic regression (LR) to identify predictors of ‘therapy response’ concerning gait speed.

**Results:**

Between 09/2017 and 09/2022, 100 (41% female) and 102 (34.3% female) PwP received GCT or PD-MCT, with significant (p < 0.003) mean or median differences (GCT vs. PD-MCT) in age (74.7 vs. 65.6 years), disease duration (9.9 vs. 7.4 years), and HY stage (3 vs. 2.5). The GCT group showed significantly reduced lower extremity (SPPB), global cognitive (MoCA) and executive function (TMT), lower quality of life, and higher fear of falling (FES-I). There were significant (p < 0.004) between-group differences in gait parameters at both normal and fast pace, e.g., reduced gait speed and step length among GCT users. After age-adjustment, differences in gait speed, fast-pace step length, lower extremity and executive function, fear of falling and quality of life persisted. The exploratory LR model was statistically significant (p < 0.05, R^2^ = 0.312) and revealed lower fear of falling and gait speed as predictors of ‘therapy response’, independent of therapy type, age, sex, disease duration or stage.

**Conclusion:**

GCT users show higher age and severity, particularly concerning mobility impairments independent of age. It is unclear if, on a national level, actual PD-MCT/GCT user groups align with intended target groups. Health insurance data analyses could help refine clinical recommendations and public health policies for more targeted multidisciplinary PD care.

**Trial registration:**

Park Move Study: DRKS, DRKS00020948. Registered 30 March 2020—retrospectively registered, https://drks.de/search/de/trial/DRKS00020948/details

**Supplementary Information:**

The online version contains supplementary material available at 10.1186/s42466-025-00409-9.

## Background

Appropriate care for people with Parkinson's disease (PD) as one of the fastest-growing neurological diseases [1] is an increasing challenge for our societies [2]. Due to the complex clinical presentation ranging from motor symptoms and gait disorders to non-motor symptoms such as cognitive and affective complaints, autonomic dysfunction, or pain [3], individual needs and therapy goals vary greatly [4]. Therefore, an individualized multidisciplinary approach to PD care has been suggested ideal to reduce symptoms and maintain quality of life sustainably [5–7]. In the sense of rehabilitation, sheer symptom control is complemented by aids to hold or restore activities of daily living, autonomy and participation [8]. Temporally, rehabilitation goals vary over the course of disease with different needs to be covered [4]. Spatially, rehabilitation is delivered in different sectors, i.e., a home-based, outpatient, day-clinic, inpatient, or integrated setting [9].

In Germany, multidisciplinary care is predominantly delivered in the inpatient sector of the hospital-focused and fragmented healthcare system [10]. In general, people with PD are more often referred to single disciplines, especially physiotherapy, than to multidisciplinary approaches [11]. Up to 42% of the ~ 420,000 people with PD in Germany receive exclusively medication and only 36% physiotherapy (PT), 6% occupational (OT), and 4% speech and language therapy (SLT), respectively, in the community [12]. Inpatient multidisciplinary interventions for people with PD in Germany are defined by reimbursement requirements from the Operation and Procedure Classification System (OPS, [13]) and include so called ‘Complex Therapies’ such as ‘PD Multimodal Complex Therapy’ (PD-MCT; OPS-8-97D) and ‘Early Rehabilitative Geriatric Complex Therapy’ (GCT; OPS-8-550). Both interventions involve an inpatient stay between 7 and 21 days or more where pharmacological therapy by board-certified physicians is complemented by individualized non-pharmacological therapies such as physiotherapy, occupational and speech and language therapy or neuropsychology [13]. They intend to optimize functional ability, reduce disability, and improve health-related quality of life. For example, therapy may include proprioceptive or gait training in physiotherapy, amplitude and fine motor skill training in occupational therapy, articulation and swallowing training in speech and language therapy, as well as drug adjustment and neuropsychological assessment. While PD-MCT is specifically offered to people with PD, GCT is more generally offered to people characterized by higher age and multimorbidity [14–16]. The interventions have shown beneficial effects on PD motor and non-motor symptoms or gait parameters in observational studies [17–25], which adds to international evidence from randomized, controlled trials investigating interventions with longer duration and higher intensity [26–29].

Irrespective of their effectiveness, the appropriate use of these inpatient multidisciplinary interventions in PD is not well defined. Appropriate care can be conceptualized as an alignment of the intended target group with the actual user group where health-related needs are covered by healthcare services like PD-MCT and GCT [30, 31] and where neither under- nor overuse takes place. Whereas there are recommendations on the use of PD-MCT relying on expert consensus from guidelines [32] or working groups [33], there is no clear guidance on the use of GCT in PD. In parallel, while users of PD-MCT have been characterized as predominantly having an age of less than 70 years, moderate to severe impairment (HY 3–4; G20.1), and motor fluctuations [34], less is known about people with PD treated with GCT. GCT case numbers increased from ~ 111,000 to ~ 370,000 between 2005 and 2023 with an unclear proportion of people with PD [35]. In 2016, ~ 3200 PD inpatients were treated at geriatric departments. Although multidisciplinary PD inpatient therapies are used widely and increasingly in Germany as shown by a nearly sevenfold increase in PD-MCT cases at more than 200 centers between 2008 and 2023 [34, 35], it has not yet been investigated whether the user groups of PD-MCT and GCT differ from one another. More knowledge could guide referrals to PD rehabilitation on an individual level and help further define the role of PD inpatient rehabilitation on a public level. Therefore, this bicenter prospective observational study aimed to compare the user groups of PD-MCT and GCT using routine clinical data from observational trials [18, 36, 37]. In an exploratory attempt, the predictors of a therapy response concerning wearable device-based gait speed as unidimensional surrogate outcome were examined.

## Methods

### Study design

This bicenter prospective observational study was a sub-analysis of the multicenter ComOn-Study [37] coordinated by UKSH University Hospital Kiel, Germany. Data from the locations Kiel and Bochum were used. The Bochum data were part of the Park-Move study [18, 36] (DRKS-ID: DRKS00020948).

### Participants

Participants were people with PD based on the diagnostic criteria of the UK Brain Bank [38] and the Movement Disorder Society (MDS) [39] who took part in a 14-day inpatient multidisciplinary complex therapy at the Department of Neurology at St. Josef-Hospital, Ruhr-University Bochum, Germany (PD-MCT), or at the Department of Neurogeriatrics at UKSH University Hospital Kiel, Germany (GCT), between 09/2017 and 09/2022. The selection criteria have been described previously [18, 37] and comprise secondary or atypical Parkinsonian syndromes as exclusion criteria, for example. Individuals with or without walking aids were included.

### Setting and procedure

Individuals with PD took part primarily, i.e. for planned complex therapy, or secondarily, e.g. after an emergency admission. Individuals were included if they met the inclusion criteria and agreed to participate.

At the beginning (T1) of the respective interventions, clinical data were collected from the participants. Gait analyses were performed at the beginning (T1) and end (T2) of the therapy using wearable devices.

### Intervention

The Operation and Procedure Classification System (OPS, [13]) details general reimbursement requirements for PD-MCT (OPS-8-97D) and GCT (OPS-8-550; Fig. 2). Both interventions require a minimum duration of 7 days. The professional teams have to be led by board-certified medical specialists (neurologists and geriatricians, respectively) and meet at weekly team meetings including documentation of individual therapy results and goals. Non-pharmacological therapies have to include at least physiotherapy (PT) and occupational therapy (OT) and take place in individual and group sessions (with higher proportions of individual sessions during GCT). The minimum therapy hours per week are specified as 7.5 (PD-MCT) and 5 h (GCT) [13]. For GCT only, treatment by specialist nurses, speech and language therapists, and (neuro)psychologists must be possible, and various social and geriatric assessments must be documented.

In this study, the Kiel cohort received GCT, while the Bochum cohort received PD-MCT. Participants underwent at least 14-day inpatient interventions (8-97D.1 or 8-550.1). Although not required by OPS and similar to the GCT in Kiel, the PD-MCT in Bochum included specialist nurses, speech and language therapists, and neuropsychologists. Both therapies are inpatient multidisciplinary complex therapies intended to optimize functional ability, reduce disability, and improve health-related quality of life. Therapy included proprioceptive training or gait analysis in physiotherapy, amplitude and fine motor skill training in occupational therapy, articulation and swallowing training in speech and language therapy, as well as drug adjustment and neuropsychological assessment, as an example. A detailed description of the therapy content has already been provided [17, 22].

### Clinical and patient-reported assessments

The participants provided basic demographic data such as age, sex and disease duration. Disease severity was determined using the modified Hoehn and Yahr scale (HY) [40, 41]. To compare the medication, we calculated the levodopa equivalent daily dose (LED) [42]. The revised version of the Unified Parkinson’s Disease Rating Scale Part 3 (MDS-UPDRS Part 3) [41, 43] and the Short Physical Performance Battery (SPPB) [44] assessed motor severity and lower extremity function, respectively. The Falls Efficacy Scale International (FES-I) [45, 46] was used to identify fear of falling, and the EuroQoL questionnaire (EQ-5D-5L) [47, 48] to quantify quality of life. In addition, the Montreal Cognitive Assessment (MoCA) [49] and the Trail Making Test (TMT) [50] were used to assess global cognitive and executive function.

### Device-based gait assessment

Three wearable devices from the CE-certified RehaGait® system (Hasomed, Magdeburg, Germany) were attached to the participants’ ankles and lower back. Under supervision, the participants performed various gait tasks. Instructions were read from a tablet by the examiner, ensuring equal instructions across participants and minimizing bias from different investigators. Usually, each exercise was performed once. However, external confounding factors, disease severity, or incorrect performance could lead to repeated measurements.

The wearable devices contained 3-axis accelerometers, gyroscopes, and compasses [51], which recorded movements and send data to the tablet via Bluetooth. Using an algorithm, the raw data from the lower back were converted into various gait parameters that have been described in detail previously [18, 36].

The data of two gait tasks were examined: Walking straight for 20 m at a normal, freely selectable speed and at a fast pace, both under single-task conditions. The analysis of these tasks has already been selected and recommended in other studies [52, 53].

### Statistical methods

IBM SPSS Statistics, version 29 and R, version 4.4.3 were used to analyze the data. Outliers and normal distribution were analyzed using boxplots and the Shapiro–Wilk test. Mean values (M) and standard deviation (SD) were calculated to describe the numerical clinical parameters, while median and interquartile range (IQR) were calculated for ordinally scaled variables. To compare the clinical data of both groups, Welch's T-test and Bayesian T-test were used. Anecdotal evidence was defined by BF_10_ < 3, moderate evidence by 3 < BF_10_ < 10, strong evidence by 10 < BF_10_ < 30, very strong evidence by 30 < BF_10_ < 100 and extreme evidence by BF_10_ > 100. For ordinal or nominal variables, the Mann–Whitney U-test or Chi-square test was used instead. P-values were corrected for multiple testing using Bonferroni adjustment (adjusted α = 0.05/n; n = number of tests), resulting in an adjusted α of 0.003 for the clinical parameters and 0.004 for the sensor parameters.

The gait parameters were checked for significant correlation with velocity using Spearman's rank correlation coefficient. Significantly correlating parameters were normalized for a velocity of 1 m/s. Given many outliers, defined as cases more than 1.5 times the interquartile range below the first or above the third quartile, the non-parametric Mann–Whitney-U-test was used to compare device-based gait parameters between groups.

To adjust for age, we performed an ANCOVA with age as covariate including all clinical and device-based data with at least interval scale. The assumptions of homoscedasticity, homogeneity of regression slopes, no outliers and normal distributed residuals for ANCOVA were partially not met, which increases the chance of a type II error.

Large effects were defined by |Cohen's d|> 0.8 or |r|> 0.5 or |partial η2|> 0.14, medium effects by 0.5 <|Cohens d|< 0.8 or 0.3 <|r|< 0.5 or 0.13 <|partial η2|< 0.06, and small effects by 0.2 <|Cohens d|< 0.5 or 0.1 <|r|< 0.3 or 0.05 <|partial η2|< 0.01.

To identify predictors of therapy response, a binomial logistic regression was performed. Using the minimal clinically important difference (MCID) of improvement in velocity during normal pace as a cutoff (8.2 cm/s; [54]), we defined a binary’therapy response’ variable as a unidimensional proxy outcome. The independent variables were determined based on content considerations and screening all variables using simple logistic regression. The model was calculated with 14 predictors, based on the number of cases. All variables were checked for multicollinearity and variables with high correlation (r > 0.8) were excluded. To calculate model quality and effect size, receiver operating characteristics (ROC) analysis (Figure S1, Additional Files 1), area under the curve (AUC) values, odds ratio, the overall percentage of accuracy, Hosmer–Lemeshow test, and Nagelkerke's R were determined.

As this is an exploratory analysis, sample size calculation and power analyses were not performed. Missing values were not imputed, instead, the affected cases were excluded from the respective analysis.

## Results

Between September 2017 and September 2022, a total of 621 patients were screened (Bochum: 200, Kiel: 421; Fig. 1). Altogether, 245 patients met the inclusion criteria and were included in the study (Bochum: 102 [PD-MCT], Kiel: 143 [GCT]). The main reasons for exclusion were atypical Parkinsonian syndromes, most advanced disease stages or other neurological diagnoses than PD (in Kiel). As this real-world study was embedded into ongoing health service activities, some assessments were carried out only on a part of the study population, leading to 17.6% of missing data. Before analysis, 43 cases with missing information on age, gender, duration of disease, or disease stage were excluded. The full analysis data set consisted of n = 202 with 102 and 100 data sets from Bochum and Kiel, respectively (Fig. 2).

**Fig. 1:**
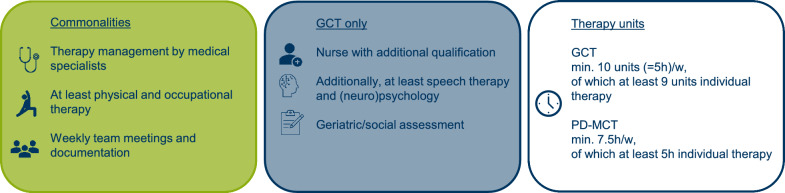
Comparison of general requirements for PD-MCT and GCT

**Fig. 2:**
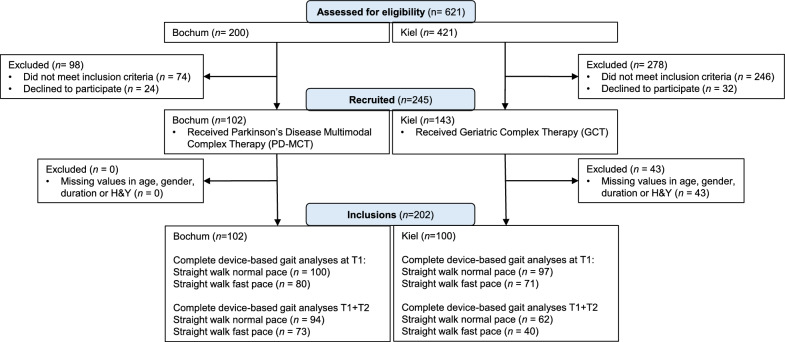
Flowchart of participants

### Demographic and clinical data

PD-MCT participants were between 48 and 86 years old (mean age 65.57 ± 9.53 years; Table 1). 34.3% of the subjects at Bochum were female. Their average disease duration was 7.44 (± 5.41) years, and the median HY stage was 2.5. GCT participants were between 48 and 91 years old (mean age 74.66 ± 6.89 years) with 41% being female. Their mean duration since diagnosis was 9.93 (± 7.22) years and the median HY stage was 3. Whereas the proportion of individuals with an HY stage of 3 or higher was 39.2% in the PD-MCT group, 93% of GCT participants had a HY stage of 3 or higher.

**Table 1:** Demographic and clinical data of PD-MCT and GCT participants

	PD-MCT			GCT			∆GCT–PD-MCT			
	n	M	SD	n	M	SD	MD	Evidence levels	p	Cohens d
Age, a	102	65.57	9.53	100	74.66	6.89	9.09	extreme	** < 0.001**	− 1.09
Sex male/female, n (%)^a^	102	67/35	(65.7/34.3)	100	59/41	(59.0/41.0)			0.403	0.07
H & Y, median (IQR)^b^	102	2.5	2	100	3	1			** < 0.001**	− 0.57
Stage, n (%)										
1		7 (6.9)			4 (4.0)					
1.5		4 (3.9)			1 (1.0)					
2		28 (27.5)			2 (2.0)					
2.5		23 (22.5)			0 (0.0)					
3		36 (35.3)			56 (56.0)					
4		4 (3.9)			37 (37.0)					
Disease duration, a	102	7.44	5.41	100	9.93	7.22	2.49	moderate	** < 0.001**	− 0.39
LED, mg	101	787.75	436.27	87	715.77	358.55	− 71.98	Evidence for H₀	0.216	0.18
MDS-UPDRS III (0–132)	102	29.25	13.55	82	33.83	15.17	4.58	anecdotal	0.034	− 0.32
SPPB (0–12)	102	8.95	2.22	87	6.18	2.17	− 2.77	extreme	** < 0.001**	1.26
FES-I (16–64)	98	25.52	9.68	77	32.17	11.14	6.65	extreme	** < 0.001**	− 0.64
EQ-5D-5L Index (0–1)	100	0.72	0.23	67	0.63	0.21	− 0.09	moderate	0.010	0.41
EQ dimensions, median (IQR)										
Mobility^b^	101	2	2	69	3	2			** < 0.001**	− 0.317
Selfcare^b^	101	1	1	69	2	2			** < 0.001**	− 0.255
Activities^b^	100	2	2	70	3	1.75			**0.002**	− 0.222
Pain^b^	101	3	1	68	3	1			0.230	− 0.084
Anxiety^b^	101	1	1	70	2	1			0.108	− 0.113
EQ-5D-5L VAS (0–100)	101	59.29	19.17	70	50.63	19.40	− 8.66	moderate	0.005	0.45
MoCA (0–30)	98	24.21	3.43	93	22.04	4.52	− 2.17	very strong	** < 0.001**	0.54
TMT B-A, s	92	57.60	46.53	74	127.34	91.30	69.74	extreme	** < 0.001**	− 0.96

Significant changes after Bonferroni-Correction (α = 0.003) are highlighted in bold

^a^Nominal scale, instead of Welch test chi-square test (significance: p-value, effect size: Cramer's V)

^b^Ordinal scale, instead of Welch test Mann–Whitney U test (significance: p-value, Effect size: Pearson correlation coefficient r)

*M* mean, *SD* standard deviation, *MD* difference of means, *Evidence levels* are based on Bayes factors (BF₁₀), reflecting the strength of evidence in favor of the alternative hypothesis, *PD-MCT* Parkinson’s Disease Multimodal Complex Therapy, *GCT* Geriatric complex therapy, *H & Y* modified Hoehn & Yahr scale, *LED* Daily Levodopa equivalent dose, *MDS-UPDRS III* Movement Disorder Society Unified Parkinson’s Disease Rating Scale Part III: motor examination, *SPPB* Short Physical Performance Battery, *FES-I* Falls Efficacy Scale International, *EQ-5D-5L* Index EuroQol 5 Dimensions-5 Level Index Value, *EQ-5D-5L VAS* EuroQol Visual Analogue Scale, *MoCA* Montreal Cognitive Assessment, *TMT B-A* Trail Making Test Part B-Part A

**Fig. 3:**
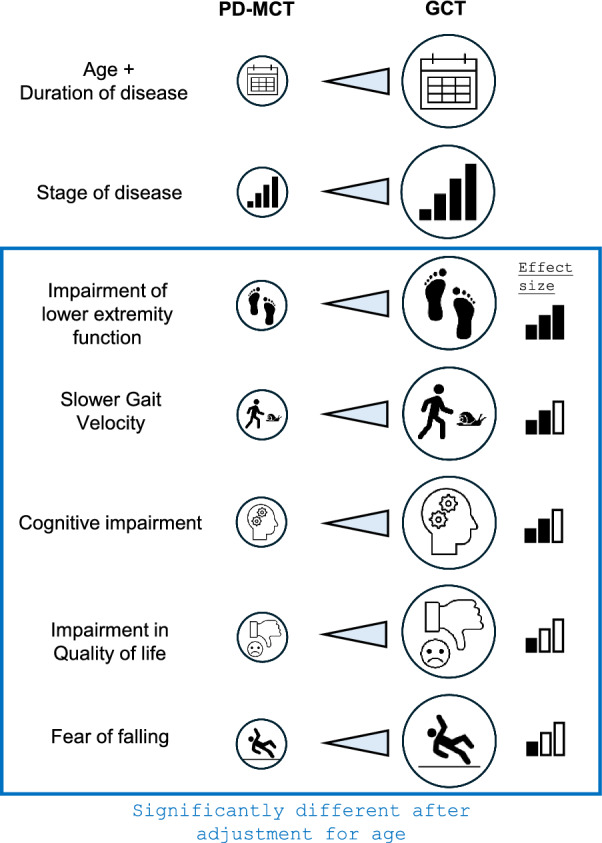
Significant differences between user groups of PD-MCT vs. GCT at baseline including an adjustment for age

Demographic and clinical variables differed significantly between groups (Table 1, Fig. 3). On average, GCT participants were older, diagnosed for a longer time, and reached higher HY disease stages. Furthermore, this group showed a lower quality of life (EQ-5D-5L) in the dimensions of self-care, mobility, and activities, inferior lower extremity function (SPPB), and more fear of falling (FES-I). Global cognitive impairment and executive dysfunction (MoCA and TMT) were also higher in the GCT group. There were no significant differences in sex distribution, LED, and motor symptoms (MDS-UPDRS III) after Bonferroni-Correction.

Particularly large effect sizes were found for age, disease stage, lower extremity function (SPPB), and executive function (TMT). Medium differences were found for the EQ mobility dimension, fear of falling (FES-I), and cognitive function (MoCA), and small differences for disease duration and the EQ-dimensions of self-care and activities.

### Device-based gait parameters

The comparison of gait characteristics between the cohorts at the beginning of the therapies (T1) at a normal and fast pace revealed significant differences (Table 2).

**Table 2:** Between-group comparison of device-based gait parameters at T1

	PD-MCT			GCT			∆GCT – PD-MCT			
	Mdn	IQR	M_Rank_	Mdn	IQR	M_Rank_	U	Z	p	r
Straight Walk Normal Pace T1	n = 100			n = 97						
Number of Steps	35.24	4.12	99.87	35.00	8.64	98.10	4763.00	− 0.22	0.829	0.02
Velocity, m/s	1.09	0.35	123.15	0.84	0.31	74.11	2435.50	− 6.04	** < 0.001**	0.43
Step length, m^a^	0.59	0.07	113.19	0.56	0.08	84.37	3431.00	− 3.55	** < 0.001**	0.25
Stride length, m^a^	1.17	0.14	113.61	1.11	0.16	83.94	3389.00	− 3.65	** < 0.001**	0.26
Cadence, steps/s	1.66	0.20	87.05	1.74	0.24	111.32	3655.00	− 2.99	**0.003**	− 0.21
Step time, s^a^	0.61	0.07	115.86	0.55	0.12	81.62	3164.00	− 4.21	** < 0.001**	0.30
Stride time, s^a^	1.20	0.13	116.65	1.10	0.26	80.80	3085.00	− 4.41	** < 0.001**	0.31
Single Limb Support, s	0.71	0.11	109.29	0.68	0.17	88.39	3821.00	− 2.57	0.010	− 0.18
Double Limb Support, s	0.44	0.06	114.26	0.41	0.09	83.27	3324.00	− 3.81	** < 0.001**	0.27
Step time variability	0.07	0.05	113.38	0.04	0.09	84.18	3412.00	− 3.59	** < 0.001**	0.25
Double Limb Support Variability^a^	0.06	0.05	111.98	0.03	0.09	85.62	3552.00	− 3.24	**0.001**	0.23
Asymmetry	0.03	0.04	94.01	0.04	0.05	104.14	4351.00	− 1.25	0.213	− 0.09

Straight Walk Fast Pace T1	n = 80			n = 71						
Number of Steps	32.90	3.09	46.15	38.71	5.08	109.63	452.00	− 8.90	** < 0.001**	− 0.63
Velocity, m/s	1.49	0.50	99.60	1.10	0.29	49.41	952.00	− 7.04	** < 0.001**	0.50
Step length, m	0.59	0.08	96.63	0.53	0.08	52.76	1190.00	− 6.15	** < 0.001**	0.43
Stride length, m	1.16	0.14	95.40	1.06	0.14	54.14	1288.00	− 5.79	** < 0.001**	0.41
Cadence, steps/s	1.76	0.22	65.08	1.84	0.26	88.31	1966.00	− 3.26	**0.001**	− 0.23
Step time, s	0.56	0.05	82.59	0.54	0.06	68.58	2313.00	− 1.96	0.049	0.14
Stride time, s	1.10	0.09	80.26	1.09	0.13	71.20	2499.00	− 1.27	0.205	0.09
Single Limb Support, s^a^	0.68	0.08	84.40	0.65	0.12	66.54	2168.00	− 2.51	0.012	− 0.20
Double Limb Support, s	0.42	0.04	83.81	0.40	0.05	67.20	2215.00	− 2.33	0.020	0.16
Step time variability^a^	0.04	0.04	69.30	0.05	0.04	83.55	2304.00	− 2.00	0.046	− 0.14
Double Limb Support Variability^a^	0.03	0.04	70.75	0.04	0.03	81.92	2420.00	− 1.57	0.118	− 0.11
Asymmetry^a^	0.02	0.02	66.59	0.03	0.05	86.61	2087.00	− 2.81	0.005	− 0.20

Significant changes after Bonferroni-Correction (α = 0.004) are highlighted in bold

^a^Distribution of both samples differ

*Mdn* median, *IQR* Interquartile range, *M*_*Rank*_ Mean Rank, *U* U-Statistic, *Z* Z-Statistic, *r* Pearson correlation coefficient, *PD-MCT* Parkinson’s Disease Multimodal Complex Therapy, *GCT* Geriatric Complex Therapy

Asked to walk at a normal pace, GCT participants walked more slowly than PD-MCT participants, with more steps per second and shorter step and stride length, needing shorter velocity-corrected times for one step or stride and double-limb support and showing lower variability. Medium effect sizes were found for velocity and stride time, whereas differences in the remaining gait parameters were small in effect.

When asked to walk at a fast pace, GCT participants needed more steps for the same distance than PD-MCT participants (large effect size), walked more slowly, with more steps per second and shorter step and stride length (all medium effect sizes).

### Between-group differences after adjusting for age

Given the large differences in age, the measurements were adjusted accordingly using ANCOVA (Table 3, Fig. 3). Even if a comparable age was assumed, GCT participants showed inferior lower extremity function (SPPB; large effect), higher executive dysfunction (TMT; medium effect), more fear of falling (FES-I; small effect) and lower quality of life (EQ-5D-5L VAS; small effect), on average. During both normal and fast pace, GCT users walked more slowly (medium to large effect). At a fast pace, they additionally took more steps, had shorter step and stride length (large effects) and higher cadence (small effect; Table 3).

**Table 3:** Between-group comparison after adjusting for age

	PD-MCT			GCT			∆GCT—PD-MCT		
	n	M	SE	N	M	SE	MD	p	partial η2
Disease duration, a	102	7.87	0.67	100	9.49	0.68	1.62	0.113	
LED, mg	101	793.26	42.73	87	709.37	46.48	− 83.90	0.212	
MDS-UPDRS III (0–132)	102	31.29	1.42	82	31.29	1.61	0.00	1.000	
SPPB (0–12)	102	8.53	0.21	87	6.68	0.23	− 1.84	** < 0.001**	0.14
FES-I (16–64)	98	26.36	1.09	77	31.10	1.25	4.75	**0.008**	0.04
EQ-5D-5L Index (0–1)	100	0.71	0.02	67	0.65	0.03	− 0.07	0.091	
EQ-5D-5L VAS (0–100)	101	59.80	2.00	70	49.89	2.45	− 9.91	**0.003**	0.05
MoCA (0–30)	98	23.36	0.39	93	22.95	0.40	− 0.41	0.495	
TMT B-A, s	92	68.79	7.34	74	113.44	8.30	44.66	** < 0.001**	0.08
*Straight Walk Normal Pace T1*									
Number of Steps	100	35.89	1.14	97	35.27	1.16	− 0.61	0.724	
Velocity, m/s	100	1.05	0.03	97	0.89	0.03	− 0.16	** < 0.001**	0.07
Step length, m	100	0.59	0.01	97	0.58	0.01	− 0.01	0.538	
Stride length, m	100	1.17	0.03	97	1.15	0.03	− 0.02	0.611	
Cadence, steps/s	100	1.69	0.02	97	1.73	0.02	0.04	0.248	
Step time, s	100	0.61	0.02	97	0.60	0.02	− 0.01	0.085	
Stride time, s	100	1.21	0.06	97	1.19	0.06	− 0.02	0.834	
Single Limb Support, s	100	0.74	0.04	97	0.74	0.04	− 0.01	0.911	
Double Limb Support, s	100	0.45	0.02	97	0.44	0.02	− 0.01	0.817	
Step time variability	100	0.09	0.01	97	0.09	0.02	− 0.01	0.757	
Double Limb Support Variability	100	0.08	0.01	97	0.08	0.01	0.00	0.856	
Asymmetry	100	0.04	0.01	97	0.06	0.01	0.02	0.087	
*Straight Walk Fast Pace T1*									
Number of Steps	80	33.03	0.43	71	39.25	0.46	6.21	** < 0.001**	0.36
Velocity, m/s	80	1.51	0.04	71	1.17	0.05	− 0.33	** < 0.001**	0.14
Step length, m	80	0.59	0.01	71	0.54	0.01	− 0.05	** < 0.001**	0.14
Stride length, m	80	1.16	0.01	71	1.07	0.01	− 0.09	** < 0.001**	0.12
Cadence, steps/s	80	1.77	0.02	71	1.85	0.02	0.07	**0.026**	0.03
Step time, s	80	0.56	0.01	71	0.55	0.01	− 0.01	0.465	
Stride time, s	80	1.10	0.01	71	1.10	0.01	0.00	0.903	
Single Limb Support, s	80	0.68	0.01	71	0.66	0.01	− 0.02	0.134	
Double Limb Support, s	80	0.42	0.00	71	0.41	0.01	− 0.01	0.274	
Step time variability	80	0.08	0.01	71	0.07	0.01	− 0.01	0.580	
Double Limb Support Variability	80	0.07	0.01	71	0.06	0.01	− 0.01	0.538	
Asymmetry	80	0.03	0.01	71	0.04	0.01	0.01	0.247	

Significant between-group differences in changes (p < 0.05) are highlighted in bold

*M* mean, *SE* standard error, *MD* difference of means

*PD-MCT* Parkinson’s Disease Multimodal Complex Therapy, *GCT* Geriatric Complex Therapy

*LED* Daily Levodopa equivalent dose,

*MDS-UPDRS III* Movement Disorder Society Unified Parkinson’s Disease Rating Scale Part III: motor examination,

*SPPB* Short Physical Performance Battery, *FES-I* Falls Efficacy Scale International,

*EQ-5D-5L Index* EuroQol 5 Dimensions-5 Level Index Value,

*EQ-5D-5L VAS* EuroQol Visual Analogue Scale, *MoCA* Montreal Cognitive Assessment,

*TMT B-A* Trail Making Test Part B—Part A

### Exploratory logistic regression model

To exploratorily determine the influence of therapy type on therapy outcome and given the scarce availability of longitudinal clinical data, an individual increase in gait velocity of ≥ 8.2 cm/s at normal pace was defined as positive ‘therapy response’ [52] as a unidimensional proxy outcome of effectiveness in a binomial logistic regression model. A positive’therapy response’ was achieved by 37% and 32% of PD-MCT and GCT participants, respectively.

The binomial logistic regression model was statistically significant, χ2(14) = 34.70, p < 0.05, with an acceptable amount of explained variance [55], as shown by Nagelkerke’s R2 = 0.312. Overall percentage of accuracy in classification was 77.0%, with a sensitivity of 53.2% and a specificity of 89.8%. The area under the curve was 0.775 (95%-CI: 0.692; 0.858) and significantly different from 0.5 (p < 0.001), meaning classification superiority of the logistic regression model over chance.

Lower fear of falling (FES-I; OR = 0.93, 95%-CI [0.88, 0.99], p = 0.014) and gait velocity (OR = 0.01, 95%-CI [0.00, 0.12], p < 0.001) at therapy start were significantly related to a positive ‘therapy response’. In contrast, type of therapy, age, sex, disease duration or stage were not significantly related to a positive ‘therapy response’ (Table S1).

## Discussion

This bicenter prospective observational study investigated the differences between user groups of two PD inpatient multidisciplinary therapies in Germany and explored whether the therapy type may impact therapy outcomes. The findings could influence referral patterns, shape the role of PD inpatient rehabilitation, and reduce morbidity.

### Group differences and therapy outcomes

GCT users were significantly older and showed longer disease duration than PD-MCT users. The mean age difference of nine years is likely due to referral patterns, as GCT targets individuals with a geriatric profile [34, 56] and those under 70 years were less frequently admitted. Real-world evidence shows that PD inpatients under 70 are more likely to receive PD-MCT [34]. The age of the PD-MCT group in this study was slightly [19, 24, 29, 57] or significantly [20, 21, 58, 59] lower than in other studies on PD-MCT, ranging from 64.1 [60] to 72.5 years [20]. This may be due to the inclusion criteria requiring sufficient ability to perform gait tasks and more rural catchment areas of some PD-MCT studies [20, 59]. GCT users with PD had similar ages to individuals included in earlier analyses of the same cohort, ranging from 72 [61] to 73 [22] years. In contrast, GCT users with any diagnosis showed mean ages between 75 [62] and 79 [63] years, indicating a need for rehabilitation at a younger age for PD patients. In previous reports, the PD duration of GCT users was around 10 years [22, 61], compared to 7.6 [60] to 9.4 [20] years for PD-MCT users. Both groups showed a higher proportion of males, reflecting the reported proportion among PD inpatients [3] but contrasting with the general 50:50 sex distribution among people with PD in Germany [12].

As a result of GCT users’ age and disease duration [64], they showed more advanced disease stages and greater cognitive impairment. Around 90% of GCT cases had a HY stage of 3 or higher, compared to roughly 40% among PD-MCT participants, demonstrating higher postural instability and fall risk [65, 66]. This is in line with previous studies that showed median HY stages of 3 [22, 61] for GCT and 2.5 [60] to 3 [20] for PD-MCT. Global cognitive impairment (MoCA < 26 [67]) was markedly more frequent in the study population than in a similarly aged German reference group (33% vs. 82% and 61% of GCT and PD-MCT users, respectively) [68]. Correspondingly, executive function as a precondition of physiological gait performance [69] was lower among GCT users.

The lower mobility-related quality of life in the GCT group may be explained by higher age [70, 71] and pronounced mobility impairments [72]. These impairments included reduced lower extremity function, slower gait velocity (which decreases with age and disease stage [73]) and shorter steps at both normal and fast pace. Interestingly, at self-selected speed, GCT participants had lower gait variability than PD-MCT users, possibly due to a higher fear of falling, leading to a more cautious, slow gait [74] and compensatory conscious movements [75]. In general, both groups walked more slowly than healthy controls [76], aligning with known reductions in velocity and step length in PD [76, 77].

While many adverse characteristics in the GCT group were age-related, higher cadence and fear of falling, and poorer gait speed, fast-pace step length, lower limb function, executive functioning, and overall quality of life persisted even after age adjustment. These pronounced mobility impairments in GCT users may be due to the higher acuteness of concurrent or PD symptoms (e.g., infections or recent falls) at admission, as the Kiel GCT primarily served emergency admissions, whereas the Bochum PD-MCT had more elective admissions from a waiting list. The GCT group may also have included PD subtypes with faster progression of gait disorder [79] and a higher load of comorbidities [80], contributing to their lower quality of life [72]. The differential pathways of non-randomized allocation to two different centers may introduce a selection bias that limits the validity and generalizability of the findings. Disentangling the drivers of differences in user groups requires data not covered by this study, such as center characteristics or social determinants of health. Nevertheless, the results are still worth discussing to inform future research using more appropriate designs.

In an exploratory analysis, therapy type did not influence a binary unidimensional ‘therapy response’, defined as at least a minimal clinically important increase in gait velocity [54], nor did group characteristics such as age or disease stage. This indicates no significant difference in gait speed effects between PD-MCT and GCT. Importantly, given the limited availability of short- and long-term clinical follow-up data and the inadequacy of a unidimensional surrogate outcome measure to reflect the multidimensional goals and pleiotropic effects of rehabilitation interventions, no definitive conclusions on benefit beyond gait speed can be drawn. Nevertheless, if one carefully interprets the absence of differential gait speed effects, one may presume similar therapy contents. As physiotherapy is probably key to gait speed improvement [78], its content and intensity may have been comparable across sites. Instead of therapy type, low fear of falling at baseline predicted gait benefits, aligning with earlier findings [22], where low fear of falling was linked to gait performance improvements after GCT. Individuals with high falls efficacy likely walked more confidently during exercises, achieving higher training intensities and greater effects [79]. Reducing fear of falling early in inpatient rehabilitation, through balance training [80] or cognitive-behavioral therapy [81], could enhance gait speed improvements. The finding that lower initial gait speed predicted its increase is consistent with earlier studies showing that worse motor symptoms or disability levels predicted improvements after PD-MCT [21, 36].

### Who uses and who should use which therapy: viewpoint and outlook

This study adds demographic, clinical and gait-related information on user groups of PD-MCT and GCT to the data reported on the provision, utilization, and effectiveness of PD-MCT from observational [17–21, 36, 57] and ecological [34] studies with less comprehensive evidence on GCT [22]. We know that a considerable number of participants have to travel to PD-MCT centers which especially applies to those with rural residence [20, 34], raising questions of disparities in access to healthcare. Further information on social determinants of PD-MCT/GCT utilization and effectiveness is not available and includes education, employment, income, access to transportation, or insurance status [82]. Additionally, whether the groups actually using these multidisciplinary PD inpatient services align with the intended target groups in need of these services, i.e. whether appropriate care without over- or under-use takes place [83, 84], cannot be determined based on current evidence. Target groups of PD-MCT or GCT are defined by recommendations from guidelines [32] and expert consensus [25, 33, 85], with no recommendations published on GCT in PD. Both interventions address a loss of physical, psychological, or social function that affects the individual’s activities of daily living. Specifically, PD-MCT should be used for crisis-like conditions, progressive clinical deterioration, complex clinical constellations and medication adjustments, or administration of device-aided therapies that are not manageable on an outpatient or day-clinic basis [16]. GCT targets acutely hospitalized individuals with higher age and multimorbidity.

Future analyses of health insurance data could assess sociodemographic determinants of PD-MCT/GCT utilization and effectiveness to refine clinical recommendations and develop public health policies ensuring appropriate care and decreasing potential health disparities on a national level, not to mention international disparities [86]. They could evaluate how safe, effective, timely, equitable, efficient and patient-centered PD inpatient rehabilitation is, following proposed quality metrics of public health [87]. Potential studies could also assess which individual indications lead to utilization of PD-MCT or GCT in the current system.

The inpatient setting for PD care carries risks such as infection, delirium [16], and high costs, and does not reflect everyday environments. Germany’s healthcare system, criticized by the OECD for its heavy reliance on hospitals, high costs, and fragmentation [10], risks losing information during transitions from inpatient to outpatient care. Benefits of PD-MCT and GCT observed during inpatient stays may diminish if there are gaps in the use of medication, allied PD healthcare, or personal exercise habits, as shown by partly regressing outcomes at follow-up [19, 60]. Alternative multidisciplinary care models, such as day-clinics [88, 89] or integrated PD care networks [90], have shown symptom reduction and cost savings [91]. Given the currently scarce availability of these services and low utilization rates of PT (36%), OT (6%), and SLT (4%) in the community [11, 12], inpatient multidisciplinary care like PD-MCT and GCT remains crucial.

In the future, PD inpatient care could be more selectively used for moderate to severe cases with subacute or acute clinical needs (HY 3–4) [92], as seen with the GCT studied here. People with mild stages (HY 1–2.5) who may still work could benefit from specialized outpatient and day-clinic care, that still need to be implemented more broadly, to prevent critical situations and delay work disability [92]. Those with the highest disease stages (HY 5), who are unfit for physical exercises during PD-MCT and GCT or live in nursing homes, may be better served by specialized outpatient neurologists through multidisciplinary palliative care networks supported by telemedicine in underserved areas.

### Generalizability and limitations

The results may inform individual and public PD management internationally. They may not apply in total to other German or international centers providing multidisciplinary PD inpatient care.

As a major limitation, the study used non-randomized allocation pathways to two centers what likely introduced a selection bias limiting validity and generalizability. In addition, a relatively large proportion of data missing for reasons of feasibility under everyday inpatient conditions (participants’ unavailability or exhaustion) may have led to decreased power and precision concerning the group comparison, possibly underestimating true values of disease severity among both groups, and to distorted parameter estimations in the regression model. Further limitations pertain to the assessment measures that were restricted in multidimensionality, ability to assess durability of effects, and ecological validity. Specifically, the use of gait speed improvement as a unidimensional surrogate outcome and the absence of clinical follow-up data warrant careful interpretation of the results that may still pave the way for further research. In addition, unsupervised mobility outcomes as ecologically more valid and patient-relevant measures would have been superior to the applied supervised gait outcomes that are prone to bias (Hawthorne effect, faster PD gait under in-hospital supervision [93]). A further relevant outcome not assessed was the number of comorbidities.

## Conclusion

GCT users are older and more severely affected than PD-MCT users, particularly in mobility impairments independent of age. No significant differences in gait speed improvement were found between the two interventions in an exploratory approach. Reducing fear of falling may enhance gait speed benefits. It is unclear if actual PD-MCT/GCT user groups align with target groups on a national level. Future analyses of health insurance data could identify determinants of PD-MCT and GCT utilization and effectiveness, refining clinical recommendations and public health policies for appropriate PD care.

## Supplementary Information

Additional file 1.

## Data Availability

The datasets used and/or analyzed during the current study are available from the corresponding author on reasonable request.

## Abbreviations

AUC: Area under the curve
EQ-5D-5L: EuroQoL questionnaire (5 dimensions, 5 levels)
FES-I: Falls Efficacy Scale-International
GCT: Geriatric early rehabilitation complex therapy
HY: Modified Hoehn & Yahr scale
IPS: Idiopathic Parkinson’ syndrome
IQR: Interquartile range
LED: Levodopa equivalent dose
M: Mean value
MDS: Movement Disorder Society
MDS-UPDRS III: Revised version of the Unified Parkinson’s Disease Rating Scale Part 3
MoCA: Montreal Cognitive Assessment
OPS: Operation and Procedure Classification System
OT: Occupational therapy
PD: Parkinson’s disease
PD-MCT: Parkinson’s Disease Multimodal Complex Therapy
PT: Physiotherapy
PwP: People with Parkinson’s disease
ROC: Receiver operating characteristics
SD: Standard deviation
SLT: Speech and Language Therapy
SPPB: Short Physical Performance Battery
TMT: Trail making test
T1: Time of assessment 1
T2: Time of assessment 2
