# Supplementary material for: User groups of inpatient multidisciplinary therapies for Parkinson’s disease in Germany: a bicenter prospective observational study
Source: Neurol Res Pract. 2025 Jul 28;7(1):53. doi: 10.1186/s42466-025-00409-9 (PMC12306091; doi:10.1186/s42466-025-00409-9)
Supplement: Supplementary file 1 — Additional file 1. [file 42466_2025_409_MOESM1_ESM.pdf]

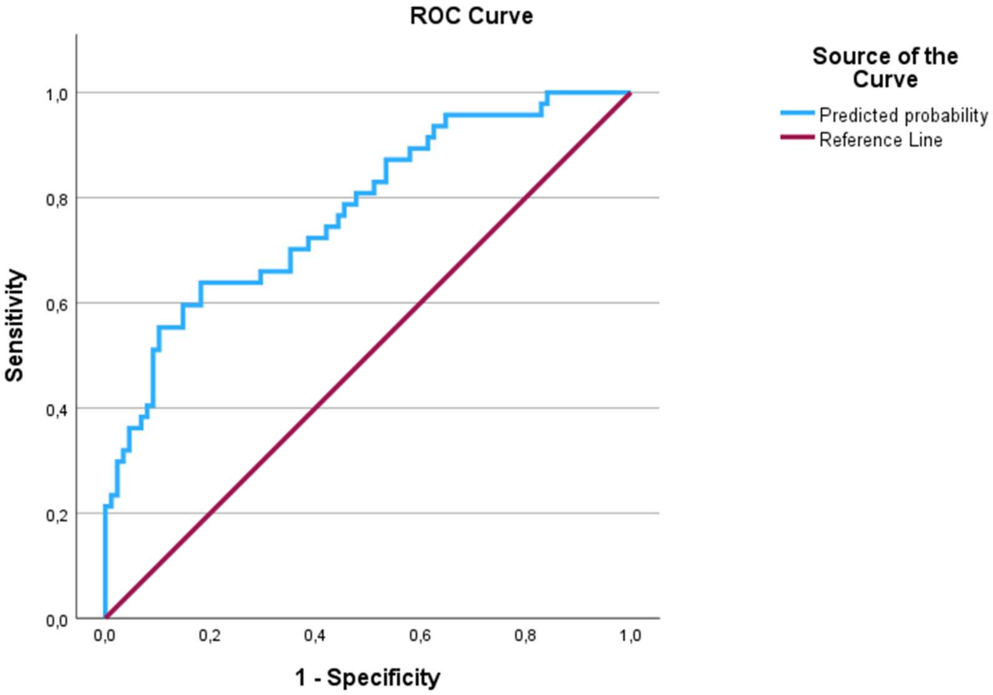

Figure S1
Receiver operating characteristics (ROC) Curve of logistic regression model

Table S1
Exploratory binomial logistic regression model: predictors of benefit in velocity after therapy

Predictors	B	SE	Wald	Df	OR	95% CI		p
						LL	UL	
Therapy	-0.58	0.54	1.17	1	0.56	0.19	1.60	0.280
Age	-0.01	0.03	0.13	1	0.99	0.94	1.04	0.714
Sex	-0.32	0.48	0.45	1	0.72	0.28	1.85	0.500
H & Y	-0.38	0.35	1.17	1	0.68	0.34	1.36	0.280
Disease duration	-0.04	0.05	0.71	1	0.96	0.88	1.05	0.399
LED	0.00	0.00	3.09	1	1.00	1.00	1.00	0.079
FES-I	-0.07	0.03	6.07	1	0.93	0.88	0.99	**0.014**
Number of steps a	0.10	0.08	1.54	1	1.10	0.94	1.29	0.215
Velocity a	-4.75	1.35	12.29	1	0.01	0.00	0.12	**<0.001**
Cadence a	-2.53	3.93	0.42	1	0.08	0.00	176.04	0.519
Step time variability a	0.30	3.25	0.01	1	1.36	0.00	789.46	0.925
Asymmetry a	6.17	5.89	1.10	1	478.33	0.00	49357911.63	0.295
Constant	8.45	9.22	0.84	1	4659.83			0.360

Significant changes ($p<0.05$) are highlighted in bold
B regression coefficient, *SE* standard error, *df* degrees of freedom, *OR* odds ratio, *CI* confidence interval, *LL/UL* lower/upper limit,
[a] for straight walk normal pace T1
H & Y modified Hoehn & Yahr scale, *LED* Daily Levodopa equivalent dose, *FES-I* Falls Efficacy Scale International
